# Ecological risk assessment of trace elements (TEs) pollution and human health risk exposure in agricultural soils used for saffron cultivation

**DOI:** 10.1038/s41598-023-31681-x

**Published:** 2023-03-20

**Authors:** Mahmoud Taghavi, Mostafa Darvishiyan, Maryam Momeni, Hadi Eslami, Reza Ali Fallahzadeh, Ahmad Zarei

**Affiliations:** 1 Department of Environment Health Engineering, School of Health, Social Determinates of Health Research Center, Gonabad University of Medical Sciences, Gonabad, Iran; 2 Student Research Committee, Gonabad University of Medical Sciences, Gonabad, Iran; 3 Department of Environmental Health Engineering, School of Health, Occupational Safety and Health Research Center, NICICO, World Safety Organization and Rafsanjan University of Medical Sciences, Rafsanjan, Iran; 4 Genetic and Environmental Adventures Research Center, School of Abarkouh Paramedicine, Shahid Sadoughi University of Medical Sciences, Yazd, Iran; 5 Department of Environment Health Engineering, School of Health, Infectious Diseases Research Center, Gonabad University of Medical Sciences, Gonabad, Iran

**Keywords:** Environmental sciences, Medical research

## Abstract

Contamination of farmland soils by trace elements (TEs) has become an international issue concerning food safety and human health risks. In the present research, the concentrations of TEs including cadmium (Cd), cobalt (Co), chromium (Cr), copper (Cu), manganese (Mn), nickel (Ni), lead (Pb), zinc (Zn) and iron (Fe) in soils of 16 farmlands were determined in Gonabad, Iran. In addition, the human health risks due to exposure to the TEs from the soils were assessed. Moreover, the soil contamination likelihood was evaluated based on various contamination indices including contamination factor $(\mathrm{CF}$), enrichment factor (EF), geo-accumulation index (Igeo), and pollution load index (PLI) calculations. The soil mean concentrations for Cd, Co, Cr, Cu, Mn, Ni, Pb, Zn and Fe ranges as 0.102, 6.968, 22.550, 29.263, 475.281, 34.234, 13.617, 54.482 and 19,683.6 mg/kg in farmland soils. The mean concentrations of the TEs decreased in the order of Fe > Mn > Zn > Ni > Cu > Cr > Pb > Co > As > Cd. Levels of all metals in this study were within the FAO/WHO and Iranian soil standards. The HQ values from investigated elements for adults and children in the studied farms were less than the limit of 1, indicating no health risks for the studied subpopulations. The results of the present research indicated no significant carcinogenic health hazards for both adults and children through ingestion, skin contact and inhalation exposure routes. $\mathrm{CF}$ values of Ni and Zn in 100% and 6.25% of farmlands were above 1, showing moderate contamination conditions. EF values of metals in farmlands were recorded as “no enrichment”, “minimal enrichment” and “moderate enrichment” classes. Furthermore, it can be concluded that the all farms were uncontaminated except Ni (moderately contaminated) based on Igeo. This is an indication that the selected TEs in the agricultural soils have no appreciable threat to human health.

## Introduction

Issues arise from the rapid process of urbanization, industrialization and land use has attracted worldwide public attention from both environmental and health perspectives^1,2^. Soil is the skin of our globe and is necessary for living organisms as it provides elements and nutrients for plants growth and serves as habitat for microflora and fauna^3^. The growing population in world significantly increase pressure on the farmlands. In order to improve the yield and profit of agricultural products, the excessive cultivation has inevitably resulted in the contamination of the soils by TEs. Farming is considered to be one of the main sources of As, Cu, Zn, Fe and Pb in the soils^4–7^. Among toxic and persistent pollutants found in agricultural soils, a special attention is paid on heavy metals. Heavy metals (including both metals and metalloids) are the most widely distributed elements of concern in soils and have been considered as priority pollutants for monitoring and controlling, and they induce threats to human health through chronic exposure by 3 routes including ingestion, inhalation and skin absorption^8–10^. Generally, the levels of TEs in farmland soils may affect the food quality, groundwater, activity of microorganisms, plant growth and yield, etc.^2,11^. The rapid trend of communities’ growth in recent decades has highlighted the need for food safety, resulting in a more use of farmlands and thus rising contamination by TEs in the agricultural soils owing to the extensive use of fertilizers, livestock manure and pesticides^12,13^. Atmospheric fallouts, and untreated sewage irrigation are other factors that can increase the levels of TEs in the soils of farmlands^14^. For instance, a study in China in 2014 showed that 19.4% of arable land across the country was contaminated by heavy metals^15^. The sources of TEs in soils include natural processes (volcanic eruptions, sea-salt sprays, forest fires, rock weathering, biogenic activities and wind-borne soil particulate matters) or human activities (mineral resource development, metal ores processing and smelting, chemical production, factory waste and wastewater irrigation)^16–19^. When high levels of TEs enter the soil environment, they destroy the structure and function of the environment and gradually deteriorate the soil quality and decrease the soil productivity and consequently affect human wellbeing via the food chain^20–23^. Heavy metals accumulated in human body have high persistence and biotoxicity, which may result in many problems such as neurotoxicity, cardiovascular disorders, cancer, kidney and bone diseases^24–29^. TEs including Cd, Pb, As and Cr are considered among the most threatening contaminants to soil quality and food security^15,30^. For instance, long-term exposure to high amounts of cadmium have detrimental effects including lung cancer, pulmonary adenocarcinomas, prostatic lesions, bone problems, kidney dysfunction, and hypertension^31,32^. Moreover, lead is an unnecessary metal for human, and ingestion of high levels of this element can harm the nervous, skeletal, circulatory, enzymatic, endocrine, and immune system of the individuals exposed to it^33^. Long-term exposure to excessive amounts of arsenic results in peripheral neuropathy, skin cancer, and peripheral vascular disease in human body^34–37^. Acute exposure to Cr causes gastrointestinal problems and even sometimes it may lead to death^38^. To diminish the cost and workload of the soil treatment effectively, identifying the possible sources of soil TEs contamination has become essential for the local authorities. TEs contamination under different types of land use induce different impacts. Soluble TEs in soils are the predominant source of TEs in plant species. Eventually, these elements may be transferred and accumulate in food crops, with the possibility of entering the food chain and accumulating in the different organs of human body^7,39–42^. The consumption of crops contaminated with poisonous TEs such as Cd, Pb, As and Cr for long term periods, even in very small amounts, constitutes health risks to individuals including depletion in immunological defenses and intrauterine growth, psychosocial dysfunctions, and many problems associated with malnutrition^43^.

An important method for estimation of the nature and likelihood of detrimental health influences in individuals exposed to noxious metals is human health risk assessment through chronic daily intake, hazard quotient, health risk index and carcinogenic risk^44^. This method has widely used by researchers to comprehensively estimate the potential hazards to human health related to exposure to several TEs^45–50^. Furthermore, in order to evaluate the status of metal contamination in agricultural soils, some geochemical indices including contamination factor (${\mathrm{C}}_{\mathrm{f}})$, enrichment factor (EF), geo-accumulation index (Igeo) and pollution load index (PLI) were employed.

Various works have estimated the risk of human exposure to TEs from soil and crops in different areas^51^. Saffron as the most expensive spice worldwide is predominantly cultivated in Gonabad. However, there is no study in literature regarding the levels of heavy metals in saffron farms in Gonabad. Therefore, a comprehensive study combining the status of TEs contamination and health risks in the farming soils across Gonabad is urgently needed in order to determine the concentrations of these priority elements for likely further pollution control and ultimately implementing management plans for reducing the associated pollution and human exposure risks. Furthermore, determination of risk contribution, identification of priority metals are also very important helping policymakers design effective management practices. Thus, the objectives of this research are to determine the levels of selective TEs and to estimate the human health risks induced by nine TEs (Cd, Co, Cr, Cu, Mn, Ni, Pb, Zn and Fe) in the soils of saffron farmlands in Gonabad city. These results can improve understanding of the human health risks of heavy metals in agricultural soils in the region. This will provide insights into contamination control, regarding human health risks and sustainable, human-friendly economic development.

## Materials and methods

### Description of study area

The study area, Gonabad city (latitude and longitude 34°20′22″ N/58°42′10″ E) with population 40,773 in 2016, is located in arid and semiarid climate region of eastern Iran in the south of Khorasan Razavi province and covers a total land area of 5789 km^2^. Its mean annual temperature is 16.5 °C and the annual mean precipitation 150 mm characterized by cold winter and hot dry summer. The annual precipitation can barely provide the needs of the irrigation water. Groundwater from the Qanats of Ghasabeh which is the world's oldest and largest networks of qanats containing 427 wells is the main source of water used for farming purpose. Saffron (*Crocus* *sativus* L.), the most expensive spice worldwide, is predominantly cultivated in Gonabad county and 3500 ha of arable land in the county is used for saffron production. Saffron is an exceptional plant in the county that is resistant to drought, and is considered a valuable product, due to the lack of water. Totally, saffron cultivation accounts for thirty five percent of the revenue from the total agricultural income of the Gonabad county. Saffron is one of the leading export products and Iran is the main supplier of saffron in the world. Industry in this city is weakly developed.

### Soil sampling and analysis

**Figure 1:**
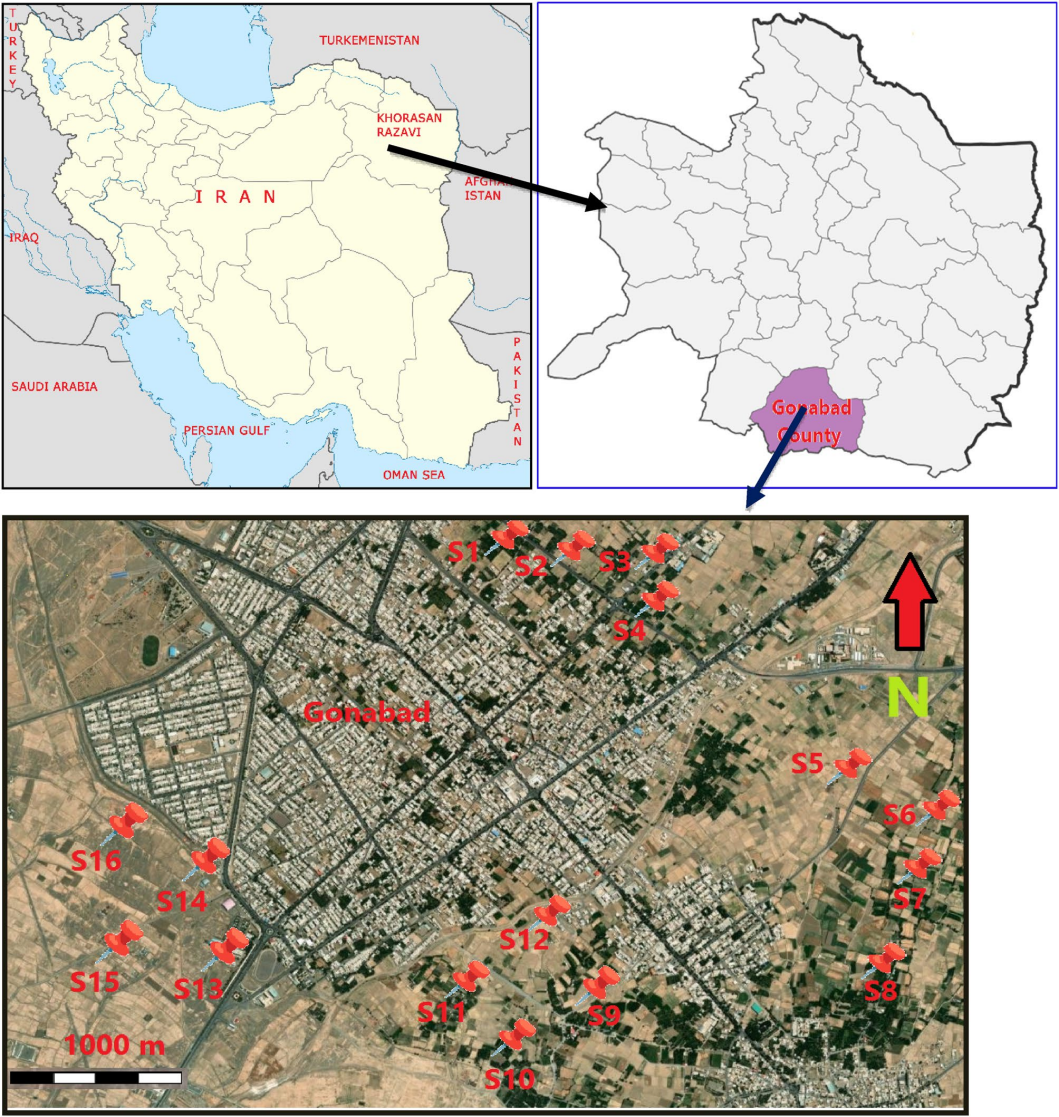
The locations of the farms where soil was sampled in Gonabad.

Field sampling was carried out in December 2021. Sixteen farms were selected for the purpose of this study to cover entire saffron farmlands of Gonabad. Totally, 16 composite soil samples (four soil samples for each farm) were taken from the topsoil (0–20 cm depth) of farms during growing season. The locations of the farms where soil was sampled in Gonabad is shown in Fig. 1. After sampling, the composite samples were stored in sealed polyethylene zipper bags, labelled and transported to the laboratory. Also, in this study, background soil samples were taken from three sites (control sites) with no farming and human activity in Gonabad and the mean metal concentrations were measured and used for the calculation of soil pollution indices. Finally, soil samples were air-dried at room temperature (23 °C) and then oven-dried at 80 °C for 72 h, ground, homogenized to remove impurities such as stones, gravel, and roots, and sieved with a two millimeters mesh sieve and placed in plastic bags until analysis. Roughly, one gram of homogenized soil samples were digested with ten millimeters of acid mixture (HNO_3_/HCL = 1/3) and then filtered through a 0.45 µm cellulose acetate filter membrane and diluted to a volume of fifty milliliters with distilled water. The total concentrations of Cd, Co, Cr, Cu, Mn, Ni, Pb, Zn and Fe were analyzed with the use of an ICP-OES (Inductively coupled plasma-optical emission spectrometry). To ensure reliability of the research results, samples were gathered and analyzed in duplicate and average levels used for the risk estimation. The LOQs of Cd, Co, Cr, Cu, Mn, Ni, Pb, Zn and Fe were 0.001, 0.007, 0.001, 0.005, 0.001, 0.05, 0.07, 0.003, and 0.003 mg/kg, respectively. The recovery values were in the 93.5–109.8% range as Cd (94.5%), Co (93.5%), Cr (95.4%), Cu (109.8%), Mn (98.5%), Ni (97.5%), Pb (79.8%) and Zn (98.1%). The concentrations of heavy metals in soil samples in the present study are reported in mg/kg dry weight basis. In this study soil pH was determined by a benchtop multiparameter analyzer (pH/Conductivity/TDS) PC820.

### Quantification of soil pollution

In order to estimate the status of pollution in agricultural soil, various pollution indices including Contamination factor, Enrichment factor, Index of geo-accumulation and Pollution load index were used as follows:

#### Contamination factor ($CF$)

The contamination factor ($\mathrm{CF}$) was first employed by Håkanson in 1980 to evaluate the enrichment of the individual heavy metals in soils in relation to their background values^52^. The following equation is used for $\mathrm{CF}$ calculation in this study:

1 $${\mathrm{C}}_{\mathrm{f}}= \frac{{\mathrm{C}}_{\mathrm{n}}}{{\mathrm{B}}_{\mathrm{v}}},$$

where Cn (in mg/kg) is the amount of the element in the soil sample and Bv (in mg/kg) is the geochemical background amount of the element in the average earth’s soil. CF is categorized as: low contamination (CF < 1); moderate contamination (1 ≤ CF < 3); considerable and significant contamination (3 ≤ CF < 6) and very high contamination (CF ≥ 6)^53^.

#### Enrichment factor (EF)

The enrichment factor (EF) is an important procedure for evaluation and discrimination of the contamination degree of TEs^54–56^. The EF value of each TE in the soil is calculated from the following equation:

2 $$\mathrm{EF}= \frac{({\mathrm{C}}_{\mathrm{n}}/{{\mathrm{C}}_{\mathrm{ref}}{)}_{\mathrm{sample }}}_{ }}{({\mathrm{B}}_{\mathrm{n}}/{{\mathrm{B}}_{\mathrm{ref}}{)}_{\mathrm{background }}}_{ }},$$

where Cn is the level of trace element n in farmland soil, ${{\mathrm{C}}_{\mathrm{ref}}}$ is the level of the reference metal, Bn is the background concentration of heavy metal n, and ${\mathrm{B}}_{\mathrm{ref}}$ is the background level of the reference element. If FE value lies between 0.5 and 1.5, it shows natural source for the metal, whereas EF > 1.5 indicates anthropogenic origin^57^. Seven categories of soil contamination are recognized based on the EF classification. EF < 1 shows no enrichment, EF < 3 is minimal enrichment, EF 3–5 is moderate enrichment, EF 5–10 is moderately severe enrichment, EF 10–25 illustrates severe enrichment, EF 25–50 shows very severe enrichment and EF > 50 reveals extremely severe enrichment^58^. In this study, mean Fe content in the soils with no human activity (control sites) of Gonabad was selected as the reference element.

### Index of geo-accumulation (${\mathrm{I}}_{\mathrm{geo}}$)

The geo-accumulation index (${\mathrm{I}}_{\mathrm{geo}}$), first introduced by Müller in 1996, has been widely employed in many studies to evaluate the probable soil pollution with TEs. It enables the estimation of soil pollution by comparing differences between present and pre-industrial levels. In the present research, the ${\mathrm{I}}_{\mathrm{geo}}$ for the soils of farming areas was calculated using Eq. (3):

3 $${\mathrm{I}}_{\mathrm{geo}}=\mathrm{Log }\left(\frac{{\mathrm{C}}_{\mathrm{n}}}{1.5{\mathrm{B}}_{\mathrm{n}}}\right),$$

where Cn is the level of target trace element present in the farming soil (in mg/kg), and B_n_ is the geochemical background value of the trace element (in mg/kg). The constant 1.5 is employed due to potential changes in a specific metal in the environment and to show small anthropogenic influences. In this work, background soil samples were taken from three sites with no farming and human activity around Gonabad and the mean trace element concentrations were used for the calculation of soil pollution indices (Table 3). The seven classes of Igeo are: ≤ 0 (uncontaminated); 0 < Igeo ≤ 1 (uncontaminated to moderately contaminated); 1 < Igeo ≤ 2 (moderately contaminated); 2 < Igeo ≤ 3 (moderately to strongly contaminated); 3 < Igeo ≤ 4 (strongly contaminated); 4 < Igeo ≤ 5 (strongly to extremely contaminated); and > 5 (extremely contaminated)^59^.

#### Pollution load index (PLI)

Pollution load index (PLI) generally assessess the level of soil contamination. This index is used to study concentrations of elements in soil samples above the reference concentration. PLI is determined using Tomlinson (1980) method as follows^60,61^:

4 $${\text{PLI}} = ({\text{CF}}_{1} \times {\text{CF}}_{2} \times \ldots \times {\text{CF}}_{{\text{n}}} )^{{(1/{\text{n}})}} ,$$

where CF is the contamination factor, n is the number of metals. Four categories of soil pollution are recognized based on the PLI classification. PLI is classified as: PLI < 1 unpolluted, 1 < PLI < 2 moderately polluted, 2 < PLI < 10 strongly polluted and PLI > 10 extremely polluted^62^.

### Human health risk assessment

In current research, the potential method expressed as the human health risk assessment obtained from US Environmental Protection Agency (USEPA) was employed to quantitatively characterize both non-carcinogenic and carcinogenic risks in adults and children groups. In this work, for the agricultural soil, 3 exposure routes were considered: (1) oral intake of soils, (2) air inhalation and (3) skin contact to soils. In order to assess health risk, first, the chronic daily intake (CDI) values of TEs through ingestion, inhalation and dermal contact routes were estimated in mg/kg.day using the formulas given in Eqs. (5)–(7) for both adults and children^63,64^. In this study, mean values of TEs were used for human health risk estimation.

The non-carcinogenic risk ($\mathrm{HQ and HI}$) though ingestion, inhalation and dermal pathways was estimated by using Eqs. (8) and (9). Similarly, the carcinogenic risk ((CR) and TCR (total cancer risk)) was also calculated by using Eqs. (10) and (11)^65,66^:

5 $${\mathrm{CDI}}_{\mathrm{ing}}={\mathrm{C}}_{\mathrm{soil}}\times \frac{\mathrm{IngR}\times \mathrm{EF}\times \mathrm{ED}}{\mathrm{BW}\times \mathrm{AT}}\times {10}^{-6},$$

6 $${\mathrm{CDI}}_{\mathrm{inh}}={\mathrm{C}}_{\mathrm{soil}}\times \frac{\mathrm{InhR}\times \mathrm{EF}\times \mathrm{ED}}{\mathrm{PEF}\times \mathrm{BW}\times \mathrm{AT}},$$

7 $${\mathrm{CDI}}_{\mathrm{derm}}={\mathrm{C}}_{\mathrm{soil}}\times \frac{\mathrm{SA}\times \mathrm{AF}\times \mathrm{ABS}\times \mathrm{EF}\times \mathrm{ED}}{\mathrm{BW}\times \mathrm{AT}}\times {10}^{-6},$$

8 $${\mathrm{HQ}}_{\mathrm{ing}/\mathrm{inh}/\mathrm{derm}}=\frac{{\mathrm{CDI}}_{\mathrm{ing}/\mathrm{inh}/\mathrm{derm}}}{{\mathrm{Rfd}}_{\mathrm{ing}/\mathrm{inh}/\mathrm{derm}}},$$

9 $$\mathrm{HI}={\sum }_{\mathrm{k}=1}^{3}\mathrm{HQ},$$

10 $${\mathrm{CR}}_{\mathrm{ing}/\mathrm{inh}/\mathrm{derm}}={\mathrm{CDI}}_{\mathrm{ing}/\mathrm{inh}/\mathrm{derm}}.\mathrm{CSF},$$

11 $$\mathrm{TCR}={\sum }_{\mathrm{k}=1}^{3}({\mathrm{CR}}_{\mathrm{ing}}+{\mathrm{CR}}_{\mathrm{inh}}+{\mathrm{CR}}_{\mathrm{derm}}),$$

where ${\mathrm{CDI}}_{\mathrm{ing}}$, ${\mathrm{CDI}}_{\mathrm{inh}}$ and ${\mathrm{CDI}}_{\mathrm{derm}}$ refers to chronic daily intake though ingestion, inhalation and skin absorption pathways expressed in mg/kg/d, respectively. ${\mathrm{C}}_{\mathrm{soil}}$ is the level of trace element in soil in mg/kg, $\mathrm{IngR}$ is the amount of ingestion rate in mg/day, EF is the exposure frequency to heavy metal in days/year, ED is the exposure duration to each heavy metal in years, BW is the body weight in kg, AT is the average time in days, $\mathrm{InhR}$ is the inhalation rate in m^3^/day, PEF is a factor relating to particulate emission in m^3^/kg, SA is the exposed skin area to pollutant in cm^2^, AF is the adherence factor of soil in (mg/cm^2^/day), ABS is dermal adsorption factor to heavy metal (unitless). ${\mathrm{HQ}}_{\mathrm{ing}}$, ${\mathrm{HQ}}_{\mathrm{derm}}$ and ${\mathrm{HQ}}_{\mathrm{inh}}$ refer to hazard quotients though ingestion, skin absorption and inhalation pathways, respectively. RfD is the reference dose of each metal in mg/kg/day. The value of RfD is the evaluation of highest allowable risk on human community through daily exposure by considering sensitive groups during a lifetime. If average CDI < RfD value, it is concluded that there would be not any detrimental health effect on human; otherwise, if CDI > RfD, it is likely that the exposure route will cause detrimental human health effects^67^. Parameters used for exposure assessment of TEs are given in Table 1. The values of RfD for the studied TEs and various exposure pathways are provided in Table 2.

**Table 1:** Input values for exposure assessment of TEs through ingestion, inhalation and dermal routes^68,69^.

Exposure items	Amount for each group		Unit
	Adults	Children	
C	–	–	mg/L
IngR	100	200	mg/day
InhR	20	7.6	m^3^/day
EF	365	365	Days/year
ED	24	6	Years
BW	70	15	Kg
AT	8760	2190	Days
SA	5700	2800	cm^2^
ABS	0.001	0.001	unitless
AF	0.07	0.2	mg/cm^3^/day
PEF	1.36 × 10^9^	1.36 × 10^9^	m^3^/kg

**Table 2:** Rfd and CSF values used for health risk assessment in this study^70–72^.

	Cd	Co	Cr	Cu	Mn	Ni	Pb	Zn	Fe
${\mathrm{RfD}}_{\mathrm{ing}}$	1.00E-03	2.00E-02	3.00E-03	4.00E-02	4.70E-02	2.00E-02	3.50E-03	3.00E-01	8.40E + 00
${\mathrm{RfD}}_{\mathrm{inh}}$	1.00E-03	5.71E-06	2.86E-05	4.02E-02	1.43E-05	2.06E-02	3.52E-03	3.00E-01	2.20E-04
${\mathrm{RfD}}_{\mathrm{derm}}$	1.00E-05	1.60E-02	5.00E-05	1.20E-02	1.84E-03	5.40E-03	5.25E-04	6.00E-02	7.00E-02
${\mathrm{CSF}}_{\mathrm{ing}}$	3.80E-03	–	5.00E-01	–	–	1.70E + 00	8.50E-03	–	–
${\mathrm{CSF}}_{\mathrm{inh}}$	6.30E + 00	–	4.20E + 01	–	–	–	4.20E-02	–	–
${\mathrm{CSF}}_{\mathrm{derm}}$	–	–	2.00E + 01	–	–	4.25E + 01	–	–	–

HI is the sum of the HQ values of a heavy metal via all the considered routes. Furthermore, ${\mathrm{CR}}_{\mathrm{ing}}$, ${\mathrm{CR}}_{\mathrm{inh}}$ and ${\mathrm{CR}}_{\mathrm{derm}}$ refers to cancer risk though ingestion, inhalation, and skin absorption pathways, respectively. $\mathrm{CSF}$ is the cancer slope factor for each studied trace element. Cancer risk of Cd, Cr, Pb and Ni are assessed in this study. ${\mathrm{CSF}}_{\mathrm{ing}}$ was based on values of 3.80E-03, 5.00E-01, 8.50E-03 and 1.70E + 00 mg/kg/day for Cd, Cr, Pb and Ni, respectively. For ${\mathrm{CSF}}_{\mathrm{inh}}$, values of 6.30E + 00, 4.20E + 01, and 4.20E-02 mg/kg/day for Cd, Cr, and Pb, respectively were used. Also ${\mathrm{CSF}}_{\mathrm{derm}}$ was based on values of 2.00E + 01, and 4.25E + 01 mg/kg/day for Cr and Ni, respectively^19,53,68,73,74^.

In this study, $\mathrm{TCR}$ is total cancer risk and estimated as the sum of CR or cancer risk values of a heavy metal via all pathways. For non-cancer risk description, if HI or HQ is equal to or greater than one, then there exists a significant probability for non-carcinogenic risk to occur^75^. In this study, for interpretation of carcinogenic risks values, the following five classification was used: very low (value < 10^–6^), low (10^–6^–10^–5^), medium (10^–5^–10^–4^), high (10^–4^–10^–3^) and very high (> 10^–3^) is of significant concern and needs further action to reduce the exposure and its associated risk^76^. Although there are uncertainties with health risk assessment methods, hence they have become important tools in estimating the relationship between human health and TEs toxicity by which we can determine both cancer and non-cancer health effects via different exposure routes^71^. The analysis of experimental data was carried out with Microsoft Office Excel and SPSS 2019.

### Ethical approval

The current manuscript is not be submitted to another journal for simultaneous consideration or publish. All the methods included in the study are in accordance with the national guidelines.

### Consent to participate

There is no human participant in the present research.

## Results and discussion

### Soil pH and occurrence of heavy metals in farmland soils

Soil pH value of a farm is a key agronomic factor that affects on biological, chemical, and physical properties and processes and eventually plant growth and yield. For example, the levels of the solubility, mobility, and bioavailability of metals in soil depend on soil pH^53^. Metals precipitates in the form of hydroxides, carbonates or insoluble organic complexes at alkaline pH values and thus the mobility of metals in soil decreases^77^. The ideal pH for most of agricultural crops should be in range from 5.5 to 7.5^78^. The values of pH measured for the studied soil samples were relatively similar, ranging from 7.2 to 7.9, which show neutral to sub-alkaline soil conditions in farmland soils of Gonabad.

Excessive application of chemicals such as fertilizers, pesticides and herbicides and also manure in agriculture owing to the need for greater crop yields and increase productivity have resulted in the contamination and accumulation of both toxic and non-toxic metals in many farmland soils^68,79,80^. Other sources of metals in soils are parent rock material, emissions from vehicles, and industrial activities^81^. This agricultural practice can lead to the enrichment of metals in soil and endanger food safety and human health. The descriptive statistics (minimum, maximum, mean and the standard deviation) for 9 TEs (Cd, Co, Cr, Cu, Mn, Ni, Pb, Zn and Fe) in the farmland soils from Gonabad are shown in Table 3. Each trace element showed a wide range of values. Cadmium levels ranged from 0.072 to 0.14 mg/kg (median 0.102 mg/kg). Cobalt ranged from 6.194 to 8.185 mg/kg (median 6.968 mg/kg). Chromium ranged from 20.25 to 25.91 mg/kg (median 22.550 mg/kg). Copper ranged from 24.325 to 35.895 mg/kg (median 29.263 mg/kg). Manganese ranged from 371.441 to 516.136 mg/kg (median 457.281 mg/kg). Nickel concentrations were in range of 32.027–37.743 mg/kg (median 34.234 mg/kg). Lead concentrations were in range of 11.628–15.527 mg/kg (median 34.234 mg/kg). Zinc concentrations were in range of 35.469–99.827 mg/kg (median 54.482 mg/kg). Concentrations of Iron were in range of 14,254.5–24,005.2 mg/kg (median 19,683.6 mg/kg). To evaluate the suitability of the soils for farming, the levels of TEs in the saffron farms were also compared with the allowable limits. Regulatory standards of TEs in agricultural soils (mg/kg) are given in Table 4. The comparison of the concentrations of heavy metals in this study with FAO/WHO and Iranian soil standards (alkaline soil standard) showed that the soils were safe for agricultural purposes in terms of TEs. As seen in Table 3, the mean concentrations of the TEs decreased in the order of Fe > Mn > Zn > Ni > Cu > Cr > Pb > Co > As > Cd.

**Table 3:** Concentrations of TEs in the soils of farmlands (n = 3).

Sampling locations	Cd	Co	Cr	Cu	Mn	Ni	Pb	Zn	Fe
L1	0.091	6.438	20.683	25.893	428.864	32.28	12.017	42.518	17,963.1
L2	0.089	6.834	22.668	24.325	371.441	32.446	13.315	45.351	18,212.6
L3	0.12	7.033	20.25	24.963	410.675	32.027	12.836	45.047	20,124.7
L4	0.072	8.104	23.555	35.895	459.816	37.743	14.609	63.849	24,005.2
L5	0.092	6.926	20.933	24.626	468.032	32.128	11.958	45.085	17,041.9
L6	0.083	8.185	25.91	28.261	486.656	37.616	14.406	51.261	23,461.8
L7	0.112	6.824	21.271	28.227	516.136	32.772	11.628	48.959	20,157.5
L8	0.084	6.74	21.683	30.689	499.158	32.816	12.177	47.553	16,277.8
L9	0.09	6.813	22.8	27.903	471.215	33.625	13.56	83.07	18,649.8
L10	0.072	6.194	23.187	32.106	459.246	33.631	13.767	35.469	21,069.9
L11	0.14	7.479	25.649	32.136	434.439	36.688	15.264	55.112	21,182.8
L12	0.12	6.977	22.082	29.479	471.171	34.699	13.808	58.604	22,485.4
L13	0.12	6.677	23.33	33.652	467.568	35.524	15.079	50.677	14,254.5
L14	0.13	6.258	21.397	31.967	441.598	32.827	15.402	50.784	19,918.3
L15	0.1	7.151	22.441	30.722	488.494	35.246	12.519	48.552	19,808.9
L16	0.12	6.848	22.961	27.371	441.986	35.679	15.527	99.827	20,323.9
Min	0.072	6.194	20.25	24.325	371.441	32.027	11.628	35.469	14,254.5
Ave	0.102	6.968	22.550	29.263	457.281	34.234	13.617	54.482	19,683.6
Max	0.14	8.185	25.91	35.895	516.136	37.743	15.527	99.827	24,005.2
STDV	0.02	0.54	1.55	3.3	34.481	1.903	1.289	15.604	2501.4
Local background values (Gonabad)	0.409	9.13	41.88	40.34	546.704	20.3	20.08	92.99	17,963.1

**Table 4:** Regulatory standards of TEs in agricultural soils (mg/kg)^4,70,83–89^.

Country/organization	Cd	Co	Cr	Cu	Mn	Ni	Pb	Zn	Fe
Australia	3	50	50	100	500	60	300	200	–
Canada	3	–	250	150	–	100	200	500	–
China	0.4	13	250	200	0.07	60	80	300	3
Germany	5	–	500	200	–	200	1000	600	–
Netherlands	13	–	180	190	–	100	530	720	–
New Zealand	3	–	290	> 0.0001	–	–	160	–	–
EU	3	–	150	140	–	75	300	300	–
UK	2	–	8	57	–	230	50	221	–
USA	3	–	400	80–200	–	72	300	200–300	–
FAO/WHO	3	50	100	100	2000	50	100	300	50,000
Iran	5	50	112	200	–	110	75	200	–

According to Table 3, the standard deviations of Fe, Mn and Zn were higher than the other studied elements, thereby showing a higher degree of dispersion. Also, Fe and Mn were found to be the most abundant elements in the studied soils. Manganese and Iron usually present naturally in relatively high concentrations in soils^82^. Therefore, slight human activities in the study area may not significantly affect the concentrations of those metals. Hence, its presence in the studied farmland soils in the area may not be attributed to any human activities neither can be considered a contaminant.

### Spearman’s correlation analysis

Spearman’s correlation matrix for TEs in soil samples from saffron farms are summarized in Table 5. Significantly positive correlation was between Ni with Co (R^2^ = 0.891), Ni with Cu (R^2^ = 0.703), Pb with Co (R^2^ = 0.662), Pb with Ni (R^2^ = 0.712), Zn with Pb (R^2^ = 0.638) and Zn with Ni (R^2^ = 0.694), suggesting that sources of these elements might be similar. In this study, strong, moderate and weak correlations are considered as those with correlation coefficients of R^2^ > 0.7, 0.5 < R^2^ < 0.7 and R^2^ < 0.5, respectively. This strong relationship indicated a common contamination source likely human activities and combustion processes for the elements. Relatively moderate positive relationship were seen between Cu with Co (R^2^ = 0.579), Pb with Cu (R^2^ = 0.509), Zn with Co (R^2^ = 0.529), Fe with Cr (R^2^ = 0.553) and Fe with Ni (R^2^ = 0.588). Cadmium, on the other hand, correlated negatively with Co, Mn, Ni and Fe. Manganese also had negative correlation with Pb and Fe.

**Table 5:** Spearman’s correlation analysis of TEs in soil samples from saffron farms.

Heavy metals	Cd	Cr	Co	Cu	Mn	Ni	Pb	Zn	Fe
Cd	1								
Cr	0.011861	1							
Co	− 0.22239	0.329412	1						
Cu	0.02817	0.008824	0.579*	1					
Mn	− 0.24167	0.111765	0.076471	0.217647	1				
Ni	− 0.04893	0.432353	0.891**	0.703**	0.211765	1			
Pb	0.329144	0.111765	0.662**	0.509*	− 0.32353	0.712**	1		
Zn	0.243151	0.385294	0.529*	0.3	0.226471	0.694**	0.638**	1	
Fe	− 0.05782	0.553*	0.464706	0.314706	− 0.00882	0.588*	0.435294	0.444118	1

*Correlation is considered significant at the 0.05 level (2-tailed) for two elements.

**Correlation is considered significant at the 0.01 level (2-tailed) for two elements.

Significant values are in bold.

### Health risk assessment

#### Non-carcinogenic risk

Due to the extensive use of fertilizers, livestock manure, and pesticides, TEs are released into the water and soil and air, harming environment and expose the communities^90^. Farmlands with contaminated soils pose health risks to the exposed population including farmers and local inhabitants^68,91^. The list of HQ and HI values are given in Table 6. As seen in the table, the HQ and HI for both adults and children have the same trends. The sum of metal HQ values for the exposure routes of for both subpopulation in the present study decrease in the order of: ingestion > inhalation > skin contact. This showed that the ingestion is a predominant route of heavy metals exposure affecting human health, then the inhalation and the skin contact is lowest. This results was also reported by a previous study^81^.

**Table 6:** Values of HQ and HI through ingestion, inhalation and dermal contact routes.

Metal	Adults			Children				HI-adult	HI-child
	HQ-ing	HQ-inh	HQ-derm	HQ-ing	HQ-inh	HQ-derm			
Cd	1.46E-04	2.15E-08	5.82E-05	1.36E-03	3.81E-08	3.82E-04		2.04E-04	1.74E-03
Co	4.98E-04	2.56E-04	2.48E-06	4.65E-03	4.55E-04	1.63E-05		7.57E-04	5.12E-03
Cr	1.07E-02	1.66E-04	2.57E-03	1.00E-01	2.94E-04	1.68E-02		1.35E-02	1.17E-01
Cu	1.05E-03	1.53E-07	1.39E-05	9.75E-03	2.71E-07	9.10E-05		1.06E-03	9.85E-03
Mn	1.39E-02	6.72E-03	1.42E-03	1.30E-01	1.19E-02	9.28E-03		2.20E-02	1.51E-01
Ni	2.45E-03	3.49E-07	3.61E-05	2.28E-02	6.19E-07	2.37E-04		2.48E-03	2.31E-02
Pb	5.56E-03	8.13E-07	1.48E-04	5.19E-02	1.44E-06	9.68E-04		5.71E-03	5.28E-02
Zn	2.59E-04	3.82E-08	5.18E-06	2.42E-03	6.77E-08	3.39E-05		2.65E-04	2.46E-03
Fe	3.35E-03	1.88E-02	1.60E-03	3.12E-02	3.33E-02	1.05E-02		2.37E-02	7.51E-02
**ΣHQ**	3.79E-02	2.59E-02	5.85E-03	3.54E-01	4.60E-02	3.83E-02	**ΣHI**	6.97E-02	4.38E-01

**Figure 2:**
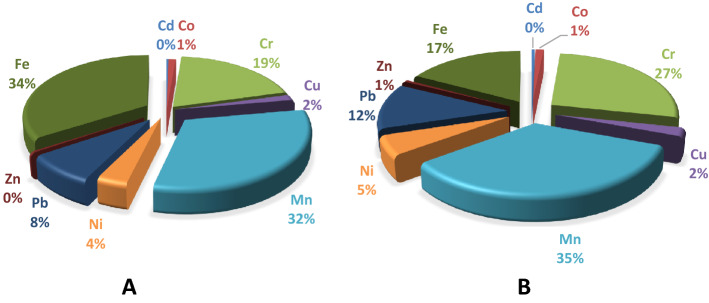
Contribution of different elements to HI for (**A**) adults and, (**B**) children.

HQ and HI values of all elements in the current study were lower than safe limit (one) for adults and children. Contribution of different elements to HI in adults and children is depicted in Fig. 2. From the figure, it can be found that Fe and Mn contributed to 34% and 35% of HI in adults and children, respectively. For adults, HI values was ranked as Fe > Mn > Cr > Pb > Ni > Cu > Co > Zn > Cd. But for children, the values of HI decreased in the order of Mn > Cr > Fe > Pb > Ni > Cu > Co > Zn > Cd.

The amounts of ΣHI both for adults and children were considerably lower than the safe limit (1). Therefore, the potential non-carcinogenic health risks for these groups can be neglected. Meanwhile, in contrast to adults, the health risk for children was higher. This may be due to their daily hand-to-mouth dietary habits and the fact that children eat more food, drink more water and beverages, and breathe more air in proportion to their body weight. This group is also more sensitive to risks induced by contaminants, because they are still growing and their bodies cannot metabolize, detoxify, and excrete toxins like adults^92^. Thus, the same contaminant concentration inducing little or no health risk to an adult might cause remarkable detrimental effects in children. This result is also reported in previous studies by researchers^71,81^. Thus, it can be concluded that children faced greater potential health hazards from the TEs of farmland soils. This finding is similar to that of a previous study^93^. Overall, exposure to a single and multiple TEs did not pose health hazard to inhabitants of this region. However, health risk of nine heavy metals were estimated and many metals which are also harmful to human health are not considered in this work. Thus, further research is suggested.

#### Carcinogenic risk

The lifetime cancer risks due to Cd, Cr, Pb and Ni in the soil of saffron farms for all the routes (ingestion, inhalation, and skin) for children up to 6 years, and adults were estimated in this work. Table 7 shows the average CR values in the soils of saffron farms. For adults, the TCR values for Cd, Cr, Pb and Ni were 6.90E-10 (very low risk), 1.66E-05 (low risk), 1.65E-07 (very low risk) and 9.14E-05 (low risk), respectively. The CR values decreased in the order of Ni > Cr > Pb > Cd for adults. For children, the TCR values for Cd, Cr, Pb and Ni were 5.42E-09 (very low risk), 1.52E-04 (medium risk), 1.54E-06 (very low risk) and 5.82E-05 (low risk), respectively and the CR values decreased in the order of Cr > Ni > Pb > Cd.

**Table 7:** CR values from agricultural soils containing metals for adults and children.

Metal	Adults			Children			CR total adults	CR total children
	CRing	CRinh	CRderm	CRing	CRinh	CRderm		
Cd	5.55E-10	1.35E-10	0.00E + 00	5.18E-09	2.40E-10	0.00E + 00	6.90E-10	5.42E-09
Cr	1.61E-05	1.99E-07	2.70E-07	1.50E-04	3.53E-07	1.77E-06	1.66E-05	1.52E-04
Pb	1.65E-07	1.20E-10	0.00E + 00	1.54E-06	2.13E-10	0.00E + 00	1.65E-07	1.54E-06
Ni	8.31E-05	0.00E + 00	8.29E-06	3.88E-06	0.00E + 00	5.43E-05	9.14E-05	5.82E-05

The results of the present study indicated no significant carcinogenic health hazards for both adults and children via ingestion, skin contact and inhalation exposure pathways in Gonabad. However, for children, chromium has typically greater potential carcinogenic health hazard in comparison to other TEs. Children are much more sensitive to carcinogenic risk from TEs exposure in soil per body weight than adults mainly because of their physiological characteristics (e.g. higher respiration rates needed per unit body weight) and behavior, i.e. their daily hand-to-mouth dietary habits. This result is similar to previous studies^53,65,71^. Similar to the non-carcinogenic risk obtained in this study, oral intake was the major exposure route contributing to the estimated cancer hazard in both subpopulations. From Table 7, it can be seen that the cancer risk decreased in the order of: CRingestion > CRdermal > CRinhalation. This finding was consistent with those reported in literature in previous studies^94,95^.

### Pollution assessment of farmlands soils

Although some TEs (e.g. zinc and copper) at low amounts are needed for normal activities of human body and other organisms, chronic direct or indirect intake of high amounts of these metals may cause health hazards. Other metals including Cd, As, Cr, Hg, Pb and Ni are toxic to the environment and human health even at low amounts^96^. In this study, the soil contamination likelihood was evaluated based on various contamination indices including contamination factor (CF), enrichment factor (EF), geo-accumulation index (Igeo), and pollution load index (PLI) calculations.

#### Contamination factor (CF)

**Figure 3:**
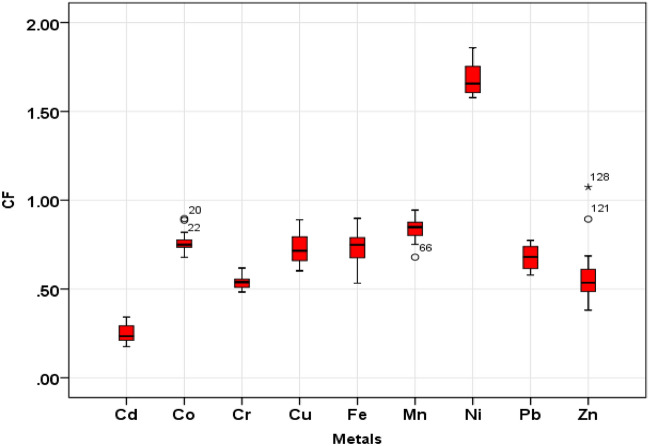
Box plots of CF values for TEs in the soils of farms.

Results for CF of elements including Cd, Co, Cr, Cu, Mn, Ni, Pb, Zn and Fe are presented in Fig. 3. Mean CF values were found in order of Ni (1.68) > Mn (0.83) > Co (0.76) > Fe (0.73) > Cu(0.72) > Pb(0.67) > Zn(0.58) > Cr(0.53) > Cd (0.24). CF values of Cd, Co, Cr, Cu, Mn, Ni, Pb, Zn and Fe in all farmlands were less than 1, showing low contamination. But CF values of Ni and Zn in 100% and 6.25% of farmlands were above 1, showing moderate contamination conditions. Generally, the results of CF showed that the quality of soil in the saffron farmlands is slightly worsened compared to the control sites.

#### Enrichment factor (EF)

**Figure 4:**
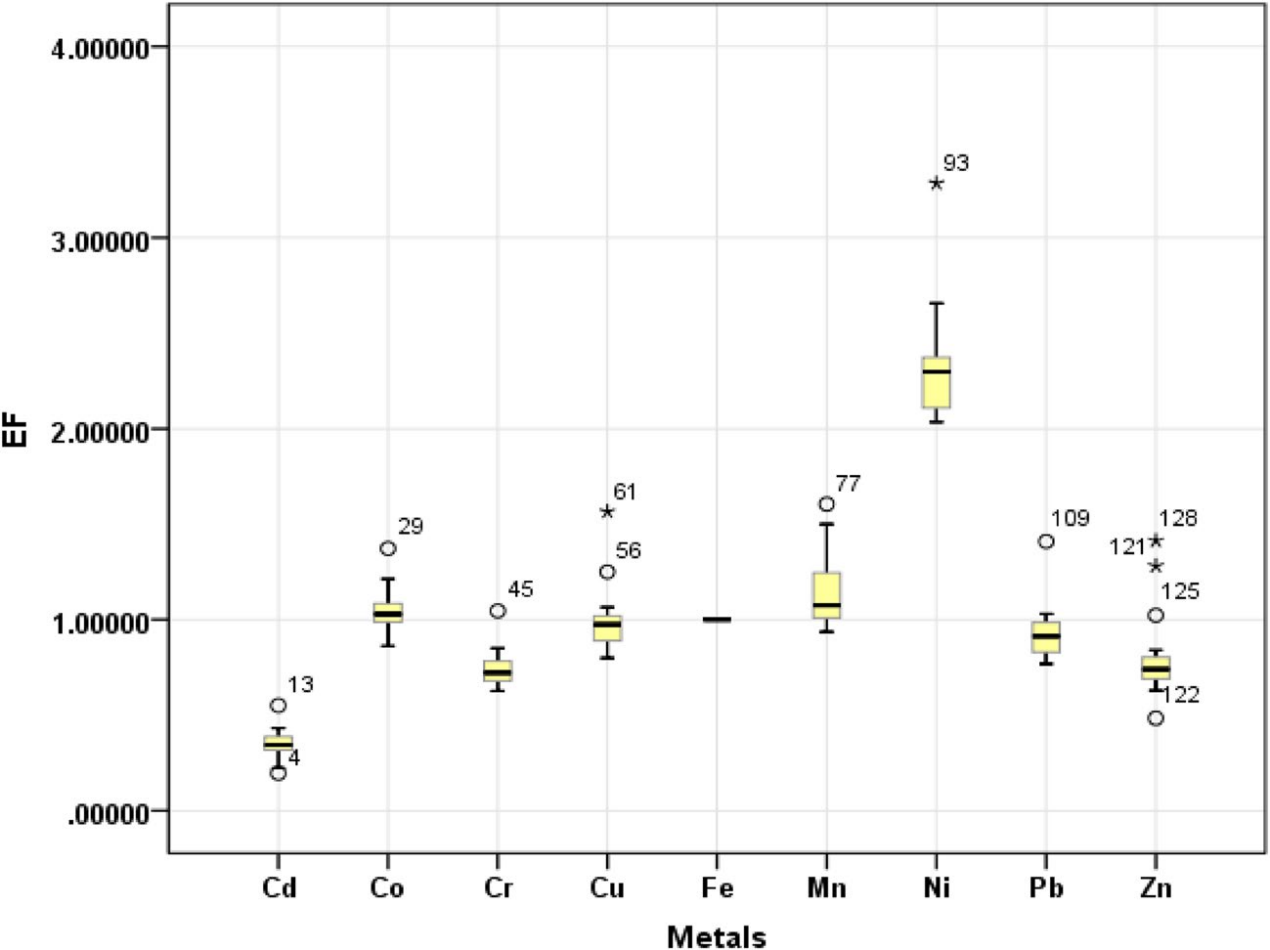
Box plots of EF values for TEs in the soils of farms.

EF values for Cd, Co, Cr, Cu, Mn, Ni, Pb, Zn and Fe are depicted in Fig. 4. The EF values obtained for Cd, Co, Cr, Cu, Mn, Ni, Pb, Zn and Fe ranged from 0.19 to 0.55 (mean 0.34), from 0.86 to 1.37 (1.04), from 0.62 to 1.04 (mean 0.74), from 0.79 to 1.56 (mean 1), from 0.93 to 1.60 (mean 1.15), from 2.03 to 3.28 (mean 2.32), from 0.76 to 1.40 (mean 0.93), from 0.48 to 1.41 (mean 0.80), and from 1 to 1 (mean 1), respectively. Mean EF values were found in the order of Ni > Mn > Co > Cu > Fe > Pb > Zn > Cr > Cd. Ni and Cd has the highest and lowest EF values in the farmlands, respectively.

EF values of Cd and Pb in all farmlands were less than 1, showing no enrichment. EF values of Co in 37.5% and 62.5% of farmlands were in “no enrichment” and “minimal enrichment” classes, respectively. For Cu, 37.5% and 62.5% of farmlands were in “minimal enrichment” and “no enrichment” classes. EF values of Mn in 18.7% and 81.3% of farmlands were in “no enrichment” and “minimal enrichment” classes. EF values of Ni for all the farmlands expect one (moderate enrichment) were in minimal enrichment class. Values of EF for Zn in 18.7% and 81.3% of farmlands were in “minimal enrichment” and “no enrichment” classes. But for Fe, all farmlands were in “minimal enrichment” class in term of EF.

#### Geo-accumulation index (Igeo)

**Figure 5:**
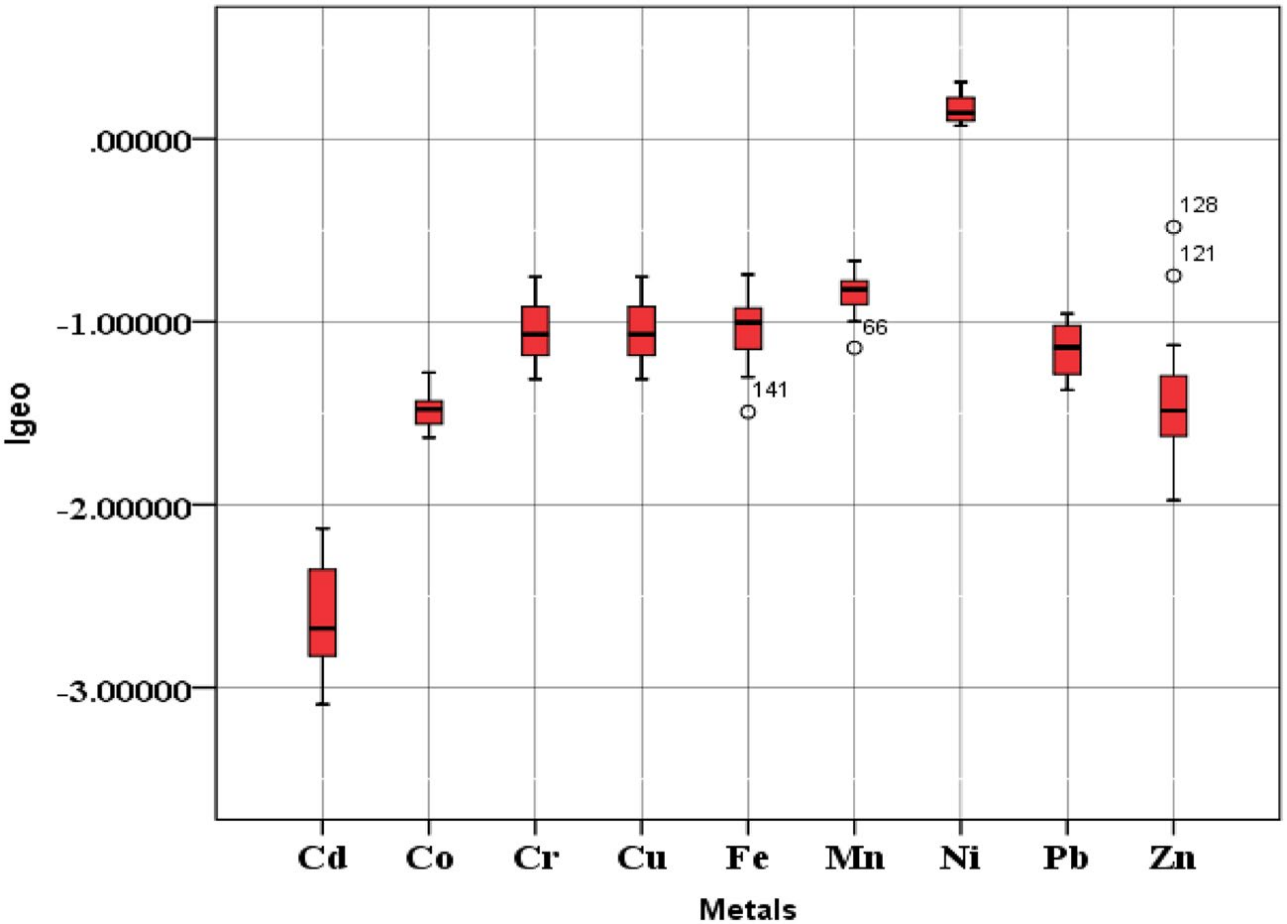
Box plots of Igeo values for TEs in the soils of farms.

Evaluation of soil contamination can also be done by comparing the measured metal levels with pre-industrial concentration levels (non-polluted areas). This method was first used by Müller (1969) to check and define metal contamination in soil and sediments^97^. Igeo index is also an indicator used to evaluate the intensity of man-made contamination. The values of Igeo for TEs in the soils of farmlands are depicted in Fig. 5. The Igeo values obtained for Cd, Co, Cr, Cu, Mn, Ni, Pb, Zn and Fe ranged from − 3.09 to − 2.13 (mean − 2.61), from − 1.14 to − 0.74 (mean − 0.97), from − 1.63 to − 1.27 (mean − 1.48), from − 1.31 to − 0.75 (mean − 1.05), from − 1.14 to − 0.66 (mean − 0.84), from 0.07 to 0.30 (mean 0.16), from − 1.37 to − 0.95 (mean − 1.15), from − 1.97 to − 0.48 (mean − 1.40), and from − 1.49 to − 0.74 (mean − 1.03), respectively. Based on the Igeo values, it can be concluded that, in terms of all metals except Ni (moderately contaminated), the soils of all farmlands belong to the class “uncontaminated”. Mean Igeo values were found in decreasing order of Ni > Mn > Co > Fe > Cu > Pb > Zn > Cr > Cd.

### Pollution load index (PLI)

**Figure 6:**
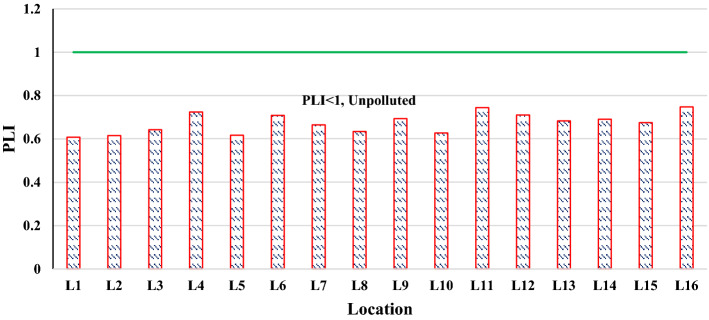
PLI values for TEs in the soils of farms.

Using Eq. (4), and PLI classification, the range of PLI values were from 0.60 to 0.74, with a mean value of 0.67; all of the farms belonged to the no pollution level with PLI < 1, according to the pollution category. PLI values for heavy metals in the soils of farms is shown in Fig. 6. Study of the contribution of different TEs to soil pollution can help develop risk mitigation strategies in the case of pollution, by adopting a prioritization of actions in agricultural soils, which could best avoid the risks for people.

## Conclusions

Farmland soil contamination by TEs is one of the most pressing issues for food safety and human health. In this study in summary, the levels of TEs in the farmland soils were estimated to evaluate the associated human health risks for inhabitants of Gonabad, Iran. By comparing the results of this study with FAO/WHO and Iranian soil standards, it can be concluded that the soils were safe for agricultural purposes in terms of TEs. The mean levels of the studied TEs decreased in the order of: Fe > Mn > Zn > Ni > Cu > Cr > Pb > Co > As > Cd. The non-carcinogenic risk of all metals fell within the recommended limit (< 1), signifying no non-carcinogenic human risk from these TEs in the farmlands. The results of the current research did not indicate significant carcinogenic health hazards for both adults and children through ingestion, skin contact and inhalation exposure pathways. However, health risk assessment also indicated that children were under higher risk in the study area. From soil indices perspective, CF values of Cd, Co, Cr, Cu, Mn, Ni, Pb, Zn and Fe in all farmlands were less than 1, showing low contamination. But CF values of Ni and Zn in 100% and 6.25% of farmlands were above 1, showing moderate contamination conditions. EF values of Cd and Pb in all farmlands were less than 1, showing no enrichment. EF values of Co in 37.5% and 62.5% of farmlands were in “no enrichment” and “minimal enrichment” classes. For Cu, 37.5% and 62.5% of farmlands were in “minimal enrichment” and “no enrichment” classes. Values of EF for Mn in 18.7% and 81.3% of farmlands were in “no enrichment” and “minimal enrichment” classes. EF values of Ni for all the farmlands expect one (moderate enrichment) were in minimal enrichment class. EF values of Zn in 18.7% and 81.3% of farmlands were in “minimal enrichment” and “no enrichment” classes. But for Fe, all farmlands were in “minimal enrichment” class in term of EF. Furthermore, it can be concluded that the all soils were uncontaminated except Ni (moderately contaminated) based on Igeo. PLI showed no contaminated conditions in the entire area.

Overall, the findings of the present work could be valuable as basic information regarding the status of heavy metal pollution in saffron farming soil as the most expensive spice in the world and health risk status regarding metals in the farms. It is anticipated that the obtained findings will serve as a baseline information for monitoring any changes in the concentrations of the heavy metals that might occur in future due to continuous agricultural activities in the farms. Also, the results of this research would be of vital help for design and implementation of contamination mitigation and remediation strategies in the saffron farmlands.

## Data Availability

All of the data analyzed and used during the current study will be available from the corresponding author (Ahmad Zarei) on reasonable request.
